# Effect of Wenshentiaojing Decoction on Hormone Level and Follicular Number in Patients with Menstrual Disorder of Polycystic Ovary Syndrome

**DOI:** 10.1155/2021/4975867

**Published:** 2021-11-29

**Authors:** Lanzhi Li, Ning Huang, Yanyan Qi, Yan Li, Lu Wang

**Affiliations:** ^1^Department of Physical Examination, Yantaishan Hospital, Yantai 264000, China; ^2^Renal Interventional Department, Affiliated Qingdao Central Hospital, Qingdao University, Qingdao 266000, China; ^3^PIVAS, Affiliated Qingdao Central Hospital, Qingdao University, Qingdao 266000, China; ^4^Interventional Department, Zhangqiu District People's Hospital, Jinan 250200, China; ^5^Department of Dermatology Laboratory, Qilu Hospital (Qingdao) Cheeloo College of Medicine, Shandong University, Qingdao 266035, China

## Abstract

**Objective:**

To explore the curative effect of Wenshentiaojing Decoction on the treatment of menstrual disorder caused by PCOS.

**Methods:**

Patients with menstrual disorders caused by PCOS admitted to our department from January 2020 to January 2021 were selected as the research objects and were divided into a control group and observation group according to the random number table method. The control group was treated with Western medicine, and the observation group was treated with Wenshentiaojing Decoction on the basis of Western medicine. The clinical efficacy of the two groups was compared. Before and after treatment, sex hormones (LH, FSH, LH/FSH, and testosterone (T)), ovarian volume, endometrial thickness, cervical mucus score, follicular number, menstrual conditions (menstrual duration, menstrual cycle, and menstrual volume), and other indicators in both groups were recorded.

**Results:**

After treatment, the total effective rate of the observation group (91.1% (41/45)) was higher than that of the control group (77.8% (35/45)), and the difference was statistically significant (*P* < 0.05). After treatment, the LH, LH/FSH, and T levels in the observation group were lower than those in the control group, while the FSH level was higher than that in the control group (*P* < 0.05). After treatment, the ovarian volume, endometrial thickness, cervical mucus score, and follicle number in the observation group were higher than those in the control group (*P* < 0.05). After treatment, the menstrual duration and menstrual volume in the observation group were longer than those in the conventional group, and the menstrual cycle was shorter than that in the conventional group (*P* < 0.05).

**Conclusion:**

For patients with menstrual disorders caused by PCOS, the treatment effect of Wenshentiaojing Decoction assisted with Western medicine is better, which can effectively improve the level of sex hormones, cervical mucus, and menstrual conditions, increase the ovarian volume, endometrial thickness, and follicle number, and improve the treatment effect, with fewer adverse reactions, which is worthy of further promotion and application.

## 1. Introduction

Polycystic ovary syndrome (PCOS) is a relatively common endocrine disease in gynecology [1, 2], mostly affecting women of reproductive age, with an incidence of about 6%–21% [3]. It is characterized by chronic anovulation, excess androgen secretion, and insulin resistance (IR). Its etiology is still unknown [4–8]. PCOS is also associated with insulin resistance in surrounding tissues, hyperinsulinemia, and abnormal degree of obesity and is one of the most common causes of menstrual irregularities in adolescent women giving birth. The menstrual cycle mainly depends on the hormone regulation of the “hypothalamic-pituitary-ovarian axis” [9–11], which is manifested as periodic endometrial exudation, and the mechanism of PCOS is the abovementioned functional abnormalities and metabolic changes [12–14]. With the change of living habits and diet structure, as well as the increase of life pressure, patients are easily affected by anxiety and other factors, resulting in a significant increase in the incidence of PCOS menstrual irregularities, which may cause reproductive disorders in patients, seriously affect their physical and mental health and quality of life, and even affect their family harmony [15, 16].

At present, the western medicine treatment of PCOS is mainly aimed at establishing a normal menstrual cycle with ovulation, restoring patients' fertility and eliminating clinical manifestations such as hirsute. It takes estrogen, progesterone symptomatic treatment. Treatment methods include the use of drugs to regulate the menstrual cycle, reduce androgen levels, improve insulin resistance, and induce ovulation, which has achieved a certain effect. However, clinical needs have not been fully met. For example, long-term hormone therapy may cause nausea, vomiting, breast tenderness, and other adverse reactions in some patients, affecting their confidence in treatment, and long-term application of hormone drugs may lead to a series of adverse reactions or drug dependence [17]. Therefore, it is particularly important to select an adjuvant therapy with high safety, strong selectivity, and fewer adverse reactions.

TCM treatment is based on the overall regulation and has the characteristics of precise curative effect and safety [18–20].Chinese medicine thinks if kidney gas is insufficient, kidney essence cannot turn unripe Qi and blood, blunt ren is not filled, blood vessel is not surplus, it and leads to menstruation maladjustment and infertility. Wenshentiaojing Decoction from “Ye Tianshi female department secret recipe for diagnosis and treatment,” is a classic formula for treating infertility, amenorrhea, irregular menstruation, and other symptoms of women. It has been widely used in ancient and modern gynecological clinical cases, with remarkable effect. In modern clinical application, according to the actual symptoms of patients, the types or dosage of medicinal materials are added or reduced, and most pills are changed into decoction, so that the therapeutic effect is remarkable. Deficiency of kidney Yang can not only stimulate the biochemical and growth of kidney Yin but also make Qi and blood run powerless, stasis and flush ren cell veins, and even lack of motive force for ovulation, so kidney deficiency is the root cause of ovulation disorder [21, 22]. Kidney deficiency can further lead to the imbalance of Qi, blood, and Yin and Yang. This fully discusses the role of the kidney in female development and reproduction [23–25]. The Wenshentiaojing decoction is a good prescription for treating infertility, amenorrhea, and irregular menstruation, which is widely used in gynecology clinic with remarkable effect. In modern clinical application according to the actual symptoms of patients, the content of each component is adjusted and pills are replaced with soup. The effect is more obvious.

This study explored the clinical efficacy of Wenshentiaojing Decoction in the adjuvant treatment of PCOS menstrual disorders and its effect on clinical symptoms and hormone levels and aimed to understand the specific situation of reducing hormone side effects of Chinese medicine decoction in the treatment of PCOS in detail and evaluate its clinical application effect and to explore the advantages of its therapeutic methods and provide reference for further promoting the application of the clinical pathway.

## 2. Materials and Methods

### 2.1. Selection of General Information and Medical Records

#### 2.1.1. Subjects

Patients with polycystic ovary syndrome complicated with menstrual disorders admitted to our department from January 2020 to January 2021 were selected as the research objects. All patients were confirmed by clinical symptoms, biochemical indicators and vaginal ultrasound examination, etc. According to the random number table method, they were divided into a control group and observation group.

#### 2.1.2. Diagnostic Criteria

1. Western diagnostic criteria: the standards recommended by the Rotterdam Expert Meeting of The European Society of Human Reproduction and Embryology and the American Society of Reproductive Medicine were adopted: ① continuous anovulation or sporadic ovulation; ② clinical manifestations of hyperandrogen and/or hyperandrogenemia. Ultrasound showed polycystic ovary. In addition, other hyperandrogen diseases such as congenital adrenal hyperplasia, Cushing's syndrome, and androgen-secreting tumors were excluded.
2. According to the diagnostic criteria of irregular menstruation in Guiding Principles for Clinical Research of New Chinese Medicine Drugs issued by the Ministry of Health of China, including premenstruation, late menstruation, irregular menstrual succession, prolonged menstruation, menorrhagia, less monthly passage, etc.
3. According to the diagnostic criteria of amenorrhea in The Guidelines for Clinical Research on New Chinese Medicine Drugs issued by the Ministry of Health of China, the former refers to primary amenorrhea, while the latter refers to secondary amenorrhea.

#### 2.1.3. Inclusion Criteria

1. Meet the diagnostic criteria of Western medicine
2. Consistent with the diagnosis of menstrual disorders or amenorrhea in traditional Chinese medicine
3. Female patients aged 18–45 years
4. Understand the effects of drugs and possible side effects, strong compliance, and active cooperation
5. The person who agrees and signs the informed consent

#### 2.1.4. Exclusion Criteria

1. Congenital adrenal hyperplasia, Cushing's syndrome, androgen-secreting tumors, and other causes of high androgen
2. Menstrual disorders caused by serious organic diseases
3. Complicated with serious cardiovascular and cerebrovascular diseases, liver and kidney injury, pulmonary diseases, blood diseases, or complicated with mental disorders
4. Pregnant or breast-feeding or postmenopausal women
5. Patients with poor compliance cannot be guaranteed to refrain from using other therapies during treatment

#### 2.1.5. Peeling Standards

1. Patients with severe adverse reactions
2. Voluntarily quit the research

### 2.2. Treatment Methods

#### 2.2.1. General Treatment

1. Resting, avoiding heavy physical activity, preventing various infections and stress states, and eating a high-protein, high-calorie diet
2. Psychological treatment: the included subjects were given psychological counseling to eliminate the interference of psychological factors, ensure a good attitude, and actively cooperate with the treatment
3. Others: doing appropriate physical exercise, but paying attention to avoid overwork

#### 2.2.2. Drug Therapy

1. Control group: clomiphene was treated. The control group began to take clomiphene for 5 consecutive days from the 5th day of menstruation, once a day, 50 mg each time. One course of treatment was 1 month, and the treatment lasted for 3 courses.
2. Treatment group: on the basis of the treatment of the control group, to warm the kidney, Tiaojing decoction Chinese medicine treatment is used.
3. The decoction is based on the addition and reduction of Yougui pill, which is combined with kidney tonifying and regulating menstruation drugs according to different menstrual cycles. Main drug composition: aconite, cinnamon, antler gum, cooked *Rehmannia officinalis*, *Cornus officinalis*, wolfberry fruit, Chinese yam, Dodder seed, *Eucommia ulmoides*, Angelica, etc.
4. Postfollicular stage: the kidney should be warmed to help Yang, nourishing qi and blood mainly. You GUI pills are added and subtracted, and donkey-hide gelatin, *Ligustrum lucidi*, Chuanduan, Dangshen, Atractylodes atractylodes, etc. are added.
5. Intermenstrual ovulation period: kidney Yijing and huoxue qi should be warmed. You GUI pills are added and subtracted, and Chuanduan, *Achyranthes bidentata*, *Salvia miltiorrhiza*, Chuanxiong, Xiangfu, etc. are added.
6. Premenstrual luteal phase: kidney should be tonified, and it should help Yang, qi, and blood circulation. Yougui pills are added and subtracted, and cistanche, *Euphorbia officinalis*, Chuanduan, Xiangfu, Chuanxiong, *Salvia miltiorrhiza*, etc. are added.
7. Menstruation period: it is advisable to warm the kidney and nourish blood, invigorate blood, and regulate menstruation. Right GUI pills are added and subtracted, and Dangshen, *Atractylodes macrocephala*, Xiangfu, Chuanxiong, *Achyranthes bidentata*, etc. are added.

Usage: one dose daily, decocted in water, divided in the morning and evening, treatment for 3 months. Medicine is taken when the fasting iodine content is too high in raw, cold, fish, mutton, and spicy products.

### 2.3. Observation Indicators

1. TCM syndrome integral scale: the TCM syndrome integral scale was formulated according to the relevant standards in The Guiding Principles for Clinical Research of Chinese Medicine New Drugs, and the TCM symptoms were quantitatively scored, and the changes of TCM syndrome integral of patients in the two groups before and after treatment were compared.
2. Test indicators: serum indicators (CRP, IGF-1, and TNF-*α*) and sex hormones (FSH, LH, and E2, T) were measured: the changes of indicators in both groups were observed before and after treatment. T lymphocytes were detected by flow cytometry: CD3+, CD4+, CD8+, and Body Mass Index (BMI).
3. Ovarian volume, endometrial thickness, cervical mucus score, and number of follicles.
4. Menstrual conditions.

### 2.4. Clinical Efficacy Standards

According to the efficacy evaluation criteria and nimodipine scoring method in The Guidelines for Clinical Research of New Chinese Medicine, the improvement of clinical symptoms of the patients was determined by the following criteria:

1. Significant effect: clinical symptoms and signs basically disappeared, menstruation basically returned to normal, B ultrasound and biochemical indicators were normal, and 70%≤ overall improvement rate <90%
2. The clinical symptoms and signs were improved compared with before treatment, menstruation basically returned to normal, B ultrasound and biochemical indicators were improved, and 30%≤ overall improvement rate <70%
3. Invalid: there were no significant changes or exacerbations in clinical symptoms, signs, and menstruation, and the overall improvement rate was less than 30%
4. The levels of sex hormones after treatment were compared between the two groups, and the observation indexes included FSH, LH, T, E2, and LH/FSH values

### 2.5. Statistical Analysis Methods

Statistical software SPSS22.0 was used for statistical analysis. Measurement data were described by mean ± standard deviation (‘*X* ± *S*'). An independent-sample *T* test was used for intergroup comparison, and a paired-sample *T* test was used for intragroup comparison. A C2 test was used for counting data. *P* < 0.05 was considered as a significant difference.

## 3. Results

### 3.1. General Data Analysis

The control group was 20–38 years old, with an average age of (28.5 ± 2.3) years. The course of disease ranged from 5 months to 8 years, with an average course of (4.7 ± 1.2) years. There were 30 married cases and 15 unmarried cases. There were 27 cases with fertility history and 18 cases without fertility history. The observation group was 20–39 years old, with an average age of (28.2 ± 2.4) years. The course of disease ranged from 6 months to 8 years, with an average course of (4.5 ± 1.1) years. 32 cases were married, and 13 cases were unmarried. There were 29 cases with fertility history and 16 cases without. There was no significant difference in general data between the two groups (*P* > 0.05), as shown in Table 1.

### 3.2. Therapeutic Efficacy Evaluation

#### 3.2.1. Comparison of Therapeutic Effects

**Figure 1:**
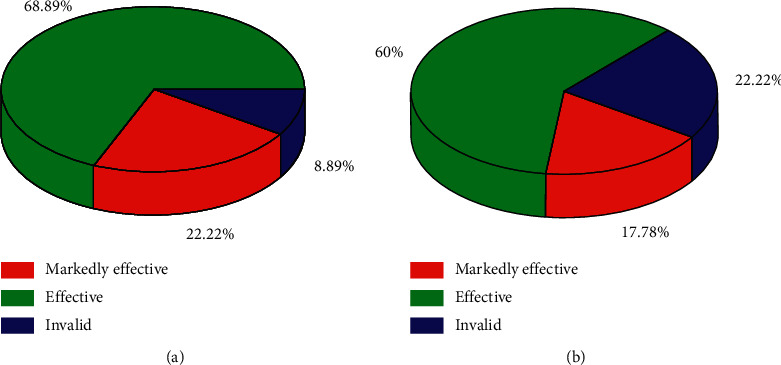
Comparison of efficacy between the two groups after treatment. (a) Observation group treatment efficacy; (b) control group treatment efficacy.

Comparison of curative effect between 2 groups. The curative effect of the observation group was significantly better than that of the control group (*X*^2^ = 2.159, *P*=0.016 < 0.05), as shown in Figure 1.

#### 3.2.2. TCM Syndrome Integral

There was no significant difference in the scores of menstrual volume syndrome, menstrual cycle syndrome, and overall TCM syndrome between the two groups before treatment (*P* > 0.05). After treatment, the scores of menstrual volume syndrome, menstrual cycle syndrome, and overall TCM syndrome in both groups were significantly lower than those before treatment (*P* < 0.05). Compared with the control group, the scores of menstrual volume syndrome, menstrual cycle syndrome, and overall TCM syndrome in the observation group were lower after treatment (*P* < 0.05), as shown in Table 2.

#### 3.2.3. Body Mass Index (BMI)

**Figure 2:**
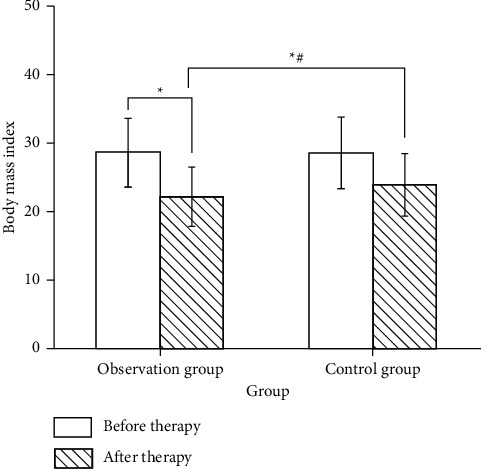
Comparison of efficacy between the two groups after treatment. *Note*. Compared with before treatment, ^*∗*^*P* < 0.05. Compared with the control group after treatment, ^#^*P* < 0.05.

Before treatment, there was no significant difference in BMI between the two groups (*P* > 0.05), indicating comparability. After treatment, the BMI of both groups decreased, and the treatment group was lower than the control group, with statistical significance (*P* < 0.05), as shown in Figure 2.

### 3.3. Comparison of Serum Indicators

**Figure 3:**
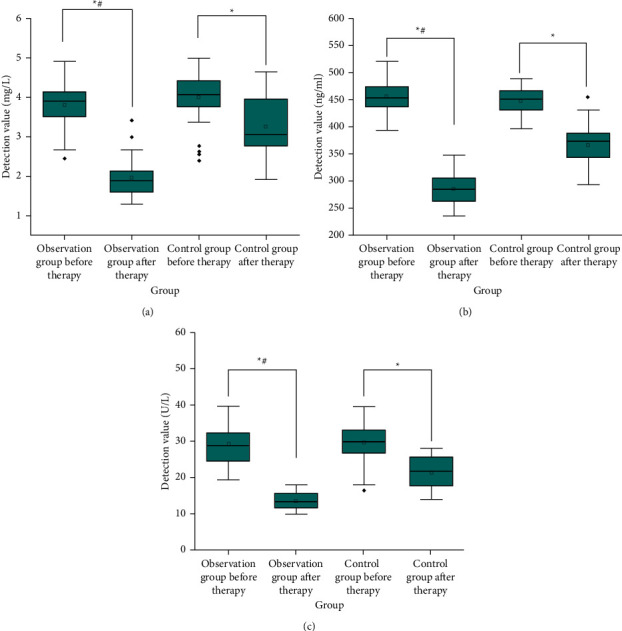
Comparison of serum CRP, IGF-1, and TNF-*α* levels between the two groups ($\bar{x}$ ± *S*). *Note*. Compared with before treatment, ^*∗*^*P* < 0.05. Compared with the control group after treatment, ^#^*P* < 0.05.

After treatment, the levels of serum CRP, IGF-1, and TNF-*α* in 2 groups were lower than those before (*P* < 0.05) Compared with the control group, the observation group improved better. After treatment, serum CRP, IGF-1, and TNF-*α* levels in the observation group were lower than those in the control group (*P* < 0.05), as shown in Figure 3.

### 3.4. Comparison of Sex Hormone Levels

Sex hormone status is a direct indicator of hypothalamic-pituitary-gonadal axis function, and abnormal sex hormone is also the key to the occurrence and development of menstrual disorders. Among them, E2 is mainly related to ovarian function, LH and FSH can synergistically regulate luteal formation and SECRETION of P, and P can induce endometrium from proliferation to secretion. Therefore, insufficient secretion of E2, LH, and FSH is an important reason for the occurrence of menstrual disorders in PCOS patients.

Before treatment, there were no significant differences in E2, FSH, and LH between 2 groups (*P* > 0.05). After treatment, hormone levels in the two groups were significantly improved (*P* < 0.05), but the levels of LH, LH/FSH, and T in the observation group were significantly lower than those in the control group (*P* < 0.05), and the levels of FSH, P, and E2 were significantly higher than those in the control group (*P* < 0.05), see Table 3 for details.

### 3.5. Comparison of Cellular Immune Indexes

**Figure 4:**
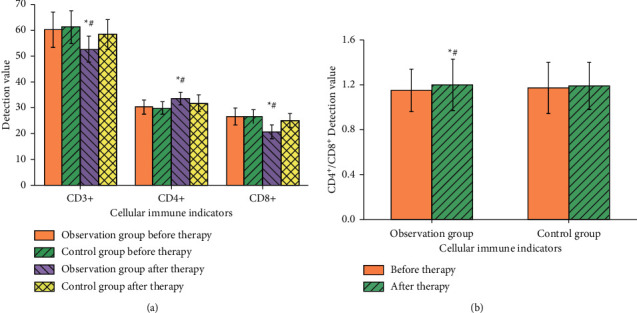
Comparison of cellular immune indexes between the two groups before and after treatment. *Note*. Compared with before treatment, ^*∗*^*P* < 0.05; compared with the control group after treatment, ^#^*P* < 0.05.

Comparison of cellular immunity and humoral immunity between the 2 groups before treatment, there was no difference in the levels of cellular immunity and humoral immunity between the 2 groups (*P* > 0.05); after treatment, the indexes of cellular immunity and humoral immunity in the observation group were improved (*P* < 0.05). Compared with the control group, the levels of CD3+ and CD8+ in the observation group decreased after treatment (*P* < 0.05), while the levels of CD4+ and CD4+/CD8+ increased (*P* < 0.05), as shown in Figure 4. The possible reason is that the chemical components of Chinese yam mainly contain diosgenin, saponin, mucilage, glycoprotein, vitamin C, etc. Pharmacological studies show that Chinese yam polysaccharide can enhance immune function and have an antiaging effect.

### 3.6. Ovarian Volume, Endometrial Thickness, Cervical Mucus Score, and Number of Follicles

In clinical practice, abnormal levels of FSH and LH in PCOS lead to abnormal follicular development, resulting in long-term anovulation, menstrual disorders, infertility, and increased incidence of endometrial cancer. Therefore, it is of great significance to observe ovarian volume and follicle number of PCOS patients.

**Figure 5:**
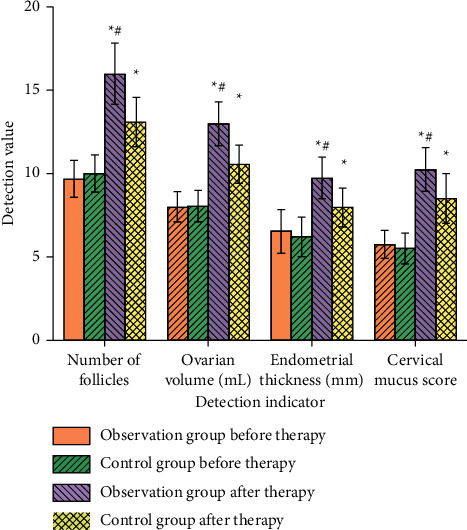
Comparison of physiological conditions between the two groups. *Note*. Compared with before treatment, ^*∗*^*P* < 0.05. Compared with the control group after treatment, ^*∗#*^*P* < 0.05.

Before treatment, there were no significant differences in ovarian volume, endometrial thickness, cervical mucus score, and follicle number between the two groups (*P* > 0.05). After treatment, ovarian volume, endometrial thickness, cervical mucus score, and follicle number in the experimental group were higher than those in the conventional group (*P* < 0.05), as shown in Figure 5. It indicates that warming kidney meridian decoction can effectively improve the number of follicles in patients. The possible reason is that *Rehmannia glutinosa* has the effect of nourishing Yin and blood, nourishing essence, and filling pulp. Wolfberry and dodder seed can nourish Yin in the kidney. *Angelica* can regulate menstruation and relieve pain and invigorate blood circulation. Modern pharmacological studies show that dodder has an estrogen-like effect and can stimulate follicular development and luteal formation. Chinese herbs such as *Angelica* for promoting blood circulation can improve microcirculation and help to improve ovarian blood supply. The combination of these drugs can improve tissue microcirculation, dilate blood vessels, improve blood oxygen supply around the lesion, and induce ovulation.

### 3.7. Menstruation

**Figure 6:**
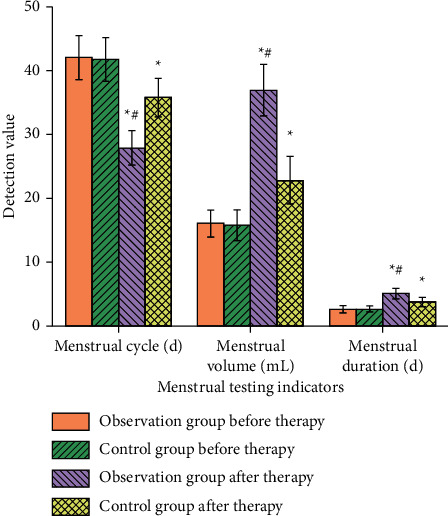
Comparison of menstrual conditions between the two groups. *Note*. Compared with before treatment, ^*∗*^*P* < 0.05. Compared with the control group after treatment, ^#^*P* < 0.05.

Before treatment, there were no significant differences in menstrual duration, menstrual cycle, and menstrual volume between the two groups (*P* > 0.05). After treatment, menstrual duration and menstrual volume in the experimental group were longer than those in the conventional group, and menstrual cycle was shorter than that in the conventional group (*P* < 0.05), as shown in Figure 6. The results showed that, after TCM-assisted treatment, the menstrual situation of the patients improved continuously, and it had statistical significance. The possible reason was that *Rehmannia glutinosa* in the prescription could rapidly increase the number of red blood cells and promote the body's hematopoietic function and blood circulation. *Angelica* can nourish blood and regulate menstruation, inhibit platelet aggregation, and inhibit the proliferation of vascular smooth muscle cells in hyperplastic intima.

## 4. Discussion

The incidence of polycystic ovary syndrome is increasing year by year, and the early clinical manifestations are often hidden. With the further development of the disease, severe menstrual disorders, loss of sexual desire, and even infertility and abortion occur. It is mainly manifested by excessive LH secretion, and the disorder of gonadotropin level secreted by the pituitary gland is very likely to lead to follicular dysplasia and hyperandrogenemia [26]. Generally, patients' condition will improve after Western medicine treatment, but some patients have poor compliance, tolerance, and side effects, and long-term alternative treatment also increases the risk of angina, myocardial infarction, osteoporosis, and fracture.

The main pathogenesis of polycystic ovary syndrome leading to menstrual disorders is the deficiency of kidney Yang and imbalance of the lung and ren, and the treatment should take warming the kidney and regulating meridian decoction as its core. According to the different menstrual cycles in our hospital, you GUI pill can be used to treat menstrual disorders, which can adjust the related indicators such as sex hormones and improve the clinical symptoms. This study evaluated the clinical efficacy of Wenshentiaojing Decoction in the treatment of menstrual disorders and explored new ideas of TCM treatment of the disease.

In this study, patients in the control group were treated with Western medicine, using clomiphene, which can improve the ovulation cycle of patients, interfere with estrogen negative feedback, and competitively inhibit estrogen receptors in the hypothalamus, which is a first-line ovulation inducing drug. Clomiphen is an estrogen-inhibiting drug, and long-term use can reduce endometrial susceptibility, seriously affect cervical mucus and endometrial thickness, increase the risk of abortion in women of childbearing age, and reduce the pregnancy rate [27]. Polycystic ovary syndrome in traditional Chinese medicine belongs to menstrual diseases, infertility and other categories, kidney deficiency, and blood stasis, and the existence of the disease is closely related.

In the treatment of menstrual disorders caused by PCOS, traditional Chinese medicine not only invigorates the liver and kidney to regulate menstruation but also promotes blood circulation and facilitates menstruation. According to syndrome differentiation, it also stimulates the function of the gonad axis in vivo, restores the positive and negative feedback function of the gonad axis, and causes the hypothalamus and pituitary gland to release FSH and LH rapidly. FSH and LH receptors on follicular granulosa cells and endometrial cells are stimulated by FSH and LH, and a large amount of estrogen (E2 and E1) is synthesized under the action of aromatase, so that all FSH and LH accumulated in the pituitary storage pool are released rapidly, forming the PEAK of FSH and LH and promoting the rapid development and maturation of follicles. In this study, the patients with menstrual disorders caused by PCOS were treated with WenshentiaoJing Decoction assisted with Western medicine, and it was found that the observation group was superior to the control group in terms of improving TCM symptoms, inflammatory factors, and sex hormones, and the total effective rate of the observation group was higher than that of the control group after treatment. LH, FSH, LH/FSH, T, ovarian volume, endometrial thickness, cervical mucus score, follicle number, and menstruation were all superior to the control group. *Achyranthes bidens*, *Salvia miltiorrhiza*, safflower, and peach kernel promote blood circulation and remove blood stasis. Duzhong and dodder strengthen the waist, knee, liver, and kidney. Antler gum, cinnamon, and aconite warm and tonify kidney Yang. Chinese yam, wolfberry fruit, cornus officinalis, and ripe *Rehmannia* root nourish the liver and spleen, nourish Yin, and nourish the kidney. Modern pharmacological studies show that *Rehmannia glutinosa* can rapidly increase the number of red blood cells and promote the body hematopoietic function and blood circulation. *Angelica* can inhibit platelet aggregation and proliferation of vascular smooth muscle cells in hyperplastic intima.

The results of this study showed that, in the observation group, TCM-assisted treatment of menstrual disorders caused by PCOS could improve the menstrual duration, menstrual cycle, and menstrual volume, without significantly increasing adverse reactions, indicating high safety. On the basis of clomiphene, the combination of warming the kidney and regulating meridian decoction, removing blood stasis, preserving the power of tonic, nourishing kidney Yang, activating blood, and tonifying kidney can further improve insulin resistance, anti-inflammatory property, improve pelvic blood microcirculation, and regulate the function of the hypothalamic-pituitary-ovarian axis.

Above all, polycystic ovary syndrome to menstrual disorder was treated by warming the kidney and regulating the menstrual function soup auxiliary treatment, patients can effectively adjust the serum sex hormone levels, and related reproductive sex gland secretion function is obviously improved, which can effectively improve the patients with cervical mucus and menstruation, restore ovarian volume, improve endometrial thickness, increase the number of follicles, and has less adverse reactions, worthy of clinical application.

## Figures and Tables

**Table 1:** Comparison of general data between the two groups ($\bar{x}$ ± *S*).

Project	Observation group (*n* = 45)	Control group (*n* = 45)	*T*	*P*
Age	20～49	20～38		
Average age	28.8 ± 2.4	28.5 ± 2.3	0.625	0.295
Course of disease	6 months–8 years	5 months–8 years		
Mean duration/year	4.5 ± 1.1	4.7 ± 1.2	0.332	0.416
Married	32	30		
Childbearing history	29	27		

**Table 2:** Comparison of TCM syndrome score difference between the two groups before and after treatment ($\bar{X}$ ± *S*).

Project	Observation group (*n* = 45)		Control group (*n* = 45)	
	Before treatment	After treatment	Before treatment	After treatment
Menstrual cycle	3.21 ± 2.31	1.58 ± 1.43^∗#^	3.56 ± 2.19	2.01 ± 1.56^*∗*^
Menstrual blood volume	3.37 ± 0.58	1.68 ± 1.62^∗#^	3.22 ± 1.78	2.17 ± 1.36^*∗*^
Dysmenorrhea	1.94 ± 1.70	1.42 ± 1.36^*∗*^	1.89 ± 1.58	1.73 ± 1.44
Inhibited sexual desire	1.84 ± 1.88	1.03 ± 1.05^∗#^	2.01 ± 1.65	1.56 ± 1.25
Doldrums	1.42 ± 1.28	0.84 ± 1.00^∗#^	2.28 ± 1.25	1.55 ± 1.09
Languid	2.68 ± 1.92	1.31 ± 1.28^∗#^	2.67 ± 0.90	1.84 ± 1.03^*∗*^
Soreness and weakness of the waist and knees	2.65 ± 1.98	1.32 ± 1.30^∗#^	2.83 ± 1.52	2.52 ± 1.01
Hypomnesis	1.73 ± 1.29	1.15 ± 1.11^∗#^	1.61 ± 1.25	2.00 ± 0.69

*Note.* Compared with before treatment, ^*∗*^*P* < 0.05. Compared with the control group after treatment, ^#^*P* < 0.05.

**Table 3:** Comparison of hormone levels between the two groups before and after treatment.

Project	Observation group (*n* = 45)		Control group (*n* = 45)	
	Before treatment	After treatment	Before treatment	After treatment
LH (mIU/L)	15.22 ± 6.22	6.28 ± 1.95^*∗*#^	15.36 ± 6.59	9.51 ± 1.62^*∗*^
FSH (mIU/L)	5.31 ± 2.39	6.50 ± 1.66^*∗*#^	5.40 ± 2.86	5.98 ± 1.62^*∗*^
LH/FSH	3.28 ± 0.93	0.94 ± 0.33^*∗*#^	3.26 ± 0.79	1.52 ± 0.61^*∗*^
T (ng/dl)	91.56 ± 16.99	48.52 ± 12.54^*∗*#^	92.05 ± 17.61	68.57 ± 18.22^*∗*^
E2 (pmol/L)	66.82 ± 15.33	157.63 ± 22.68^*∗*#^	66.92 ± 17.45	99.57 ± 18.94^*∗*^

*Note.* Compared with before treatment, ^*∗*^*P* < 0.05. Compared with the control group after treatment, ^*∗#*^*P* < 0.05.

## Data Availability

All figures and tables are included in this article.

## References

[1] Solomon C. G. (1999). The epidemiology of polycystic ovary syndrome. *Endocrinology and Metabolism Clinics of North America*.

[2] Essah P. A., Nestler J. E., Carmina E. (2008). Differences in dyslipidemia between American and Italian women with polycystic ovary syndrome. *Journal of Endocrinological Investigation*.

[3] Belenkaia L. V., Lazareva L. M., Walker W., Lizneva D. V., Suturina L. V. (2019). Criteria, phenotypes and prevalence of polycystic ovary syndrome. *Minerva Ginecologica*.

[4] Lakkakula B. V. K. S., Thangavelu M., Godla U. R. (2013). Genetic variants associated with insulin signaling and glucose homeostasis in the pathogenesis of insulin resistance in polycystic ovary syndrome: a systematic review. *Journal of Assisted Reproduction and Genetics*.

[5] Ong M., Peng J., Jin X., Qu X. (2017). Chinese herbal medicine for the optimal management of polycystic ovary syndrome. *The American Journal of Chinese Medicine*.

[6] Rajiwade S. R., Sagili H., Soundravally R., Subitha L. (2018). Endocrine abnormalities in adolescents with menstrual disorders. *Journal of Obstetrics & Gynaecology of India*.

[7] Lanham M. S. M., Lebovic D. I., Domino S. E. (2006). Contemporary medical therapy for polycystic ovary syndrome. *International Journal of Gynecology & Obstetrics*.

[8] Legro R. S., Brzyski R. G., Diamond M. P. (2014). Letrozole versus clomiphene for infertility in the polycystic ovary syndrome. *New England Journal of Medicine*.

[9] Legro R. S., Barnhart H. X., Schlaff W. D. (2007). Clomiphene, metformin, or both for infertility in the polycystic ovary syndrome. *New England Journal of Medicine*.

[10] Orio F., Muscogiuri G., Giallauria F. (2016). Oral contraceptives versus physical exercise on cardiovascular and metabolic risk factors in women with polycystic ovary syndrome: a randomized controlled trial. *Clinical Endocrinology*.

[11] Ruan X., Seeger H., Mueck A. O. (2012). Breast cancer risk during hormone therapy: experimental versus clinical data. *Minerva Endocrinologica*.

[12] Dumesic D. A., Lobo R. A. (2013). Cancer risk and PCOS. *Steroids*.

[13] Shi Y., Guo M., Yan J. (2007). Analysis of clinical characteristics in large-scale Chinese women with polycystic ovary syndrome. *Neuroendocrinology Letters*.

[14] Rostami Dovom M., Ramezani Tehrani F., Djalalinia S., Cheraghi L., Behboudi Gandavani S., Azizi F. (2016). Menstrual cycle irregularity and metabolic disorders: a population-based prospective study. *PLoS One*.

[15] Stegeman B. H., De Bastos M., Rosendaal F. R. (2013). Different combined oral contraceptives and the risk of venous thrombosis: systematic review and network meta-analysis. *BMJ*.

[16] Ratts V. S., Pauls R. N., Pinto A. B., Kraja A., Williams D. B., Odem R. R (2007). Risk of multiple gestation after ovulation induction in polycystic ovary syndrome. *Journal of Reproductive Medicine*.

[17] Ren J.-l., Zhang A.-H., Wang X.-J. (2020). Traditional Chinese medicine for COVID-19 treatment. *Pharmacological Research*.

[18] Jiang Z., Gao W., Huang L. (2019). Tanshinones, critical pharmacological components in salvia miltiorrhiza. *Frontiers in Pharmacology*.

[19] Li Z.-M., Xu S.-W., Liu P.-Q. (2018). Salvia miltiorrhiza Burge (Danshen): a golden herbal medicine in cardiovascular therapeutics. *Acta Pharmacologica Sinica*.

[20] Saraf R. S., Datta A., Sima C., Hua J., Lopes R., Bittner M. (2018). An in-silico study examining the induction of apoptosis by cryptotanshinone in metastatic melanoma cell lines. *BMC Cancer*.

[21] Yu Z., Lv H., Han G., Ma K. (2016). Ethosomes loaded with cryptotanshinone for acne treatment through topical gel formulation. *PLoS One*.

[22] Du Y., Du L., He Z., Zhou J., Wen C., Zhang Y. (2019). Cryptotanshinone ameliorates the pathogenesis of systemic lupus erythematosus by blocking T cell proliferation. *International Immunopharmacology*.

[23] Luo H., Vong C. T., Chen H. (2019). Naturally occurring anti-cancer compounds: shining from Chinese herbal medicine. *Chinese Medicine*.

[24] Qi P., Li Y., Liu X. (2019). Cryptotanshinone suppresses non-small cell lung cancer via microRNA-146a-5p/EGFR Axis. *International Journal of Biological Sciences*.

[25] Zhang J., Wen G., Sun L. (2018). Cryptotanshinone inhibits cellular proliferation of human lung cancer cells through downregulation of IGF-1R/PI3K/Akt signaling pathway. *Oncology Reports*.

[26] Sanchez N. (2018). Suitability of the national health care surveys to examine behavioral health services associated with polycystic ovary syndrome. *The Journal of Behavioral Health Services & Research*.

[27] Moulana M. (2019). Immunophenotypic profile of leukocytes in hyperandrogenemic female rat an animal model of polycystic ovary syndrome. *Life Sciences*.

